# A New Histology-Based Prognostic Index for Acute Lymphoblastic Leukemia: Preliminary Results of the “ALL Urayasu Classification”

**DOI:** 10.3390/jcm15020768

**Published:** 2026-01-17

**Authors:** Toru Mitsumori, Hideaki Nitta, Haruko Takizawa, Hiroko Iizuka-Honma, Chiho Furuya, Suiki Maruo, Maki Fujishiro, Shigeki Tomita, Akane Hashizume, Tomohiro Sawada, Kazunori Miyake, Mitsuo Okubo, Yasunobu Sekiguchi, Masaaki Noguchi

**Affiliations:** 1 Department of Hematology, Juntendo University Urayasu Hospital, 2-1-1 Tomioka, Urayasu-shi 279-0021, Japan; t.mitsumori.db@juntendo.ac.jp (T.M.); nitta@juntendo.ac.jp (H.N.); takizawa@juntendo.ac.jp (H.T.); hiiduka@juntendo.ac.jp (H.I.-H.); c-furuya@juntendo.ac.jp (C.F.); s.maruo.hf@juntendo.ac.jp (S.M.); 2 Division of Hematology, Juntendo University Juntendo Hospital, Tokyo 113-0033, Japan; 3 Institute for Environmental and Gender-Specific Medicine, Juntendo University Urayasu Hospital, Chiba 279-0021, Japan; mfujishi@juntendo.ac.jp; 4 Department of Diagnostic Pathology, Kasukabe Medical Center, Kasukabe 344-8588, Japan; sstomita@juntendo-urayasu.jp; 5 Department of Diagnostic Pathology, Juntendo University Urayasu Hospital, Chiba 279-0021, Japan; akane@juntendo-urayasu.jp; 6 Department of Clinical Laboratory, Juntendo University Urayasu Hospital, Chiba 279-0021, Japan; sawada@juntendo-urayasu.jp; 7 Department of Clinical Laboratory, Faculty of Medical Sciences, Juntendo University, Tokyo 113-8421, Japan; cpm@juntendo.ac.jp; 8 Laboratory of Blood Transfusion, Juntendo University Urayasu Hospital, Chiba 279-0021, Japan; mi-okubo@juntendo.ac.jp; 9 Hematology Clinic, Saitama Cancer Center, Saitama 362-0806, Japan; yasu_sek@saitama-pho.jp

**Keywords:** ALL, Urayasu classification, prognostic index, MRP1, AKR1B10, AKR1B1 AR1C3, CYP3A4, MDR1, ENT1

## Abstract

**Background/Objectives**: Mechanisms underlying treatment resistance in hematopoietic malignancies such as acute lymphoblastic leukemia (ALL) include (1) enhanced activity of anticancer drug efflux mechanisms (MRP1); (2) suppressed activity of anticancer drug influx mechanisms (ENT-1); (3) enhanced drug detoxification activity (AKR1B10, AKR1C3, CYP3A4); (4) influence of the tumor microenvironment (GRP94), etc. We conducted this study to comprehensively and clinically examine treatment resistance due primarily to a decrease in the tumor intracellular anticancer drug concentrations. **Methods**: The subjects were 19 ALL patients who underwent initial induction therapy with alternating Hyper CVAD/MA therapy. Antibodies against 23 types of treatment resistance-associated proteins were used for immunohistochemical analysis of tumor specimens obtained from the patients, and correlations between the results of immunohistochemistry and the overall survival (OS) were retrospectively analyzed using the Kaplan–Meier method. **Results**: Based on the patterns of expression of the enzymes involved in treatment resistance, we classified the patients (Urayasu classification for ALL, which we believe would be very useful for accurately stratifying patients with ALL according to the predicted prognosis), as follows: Good prognosis group, *n* = 1, 5%: AKR1B1(+)/AKR1B10(−), 5-year overall survival (OS), 100%; Intermediate prognosis-1 group, *n* = 9, 5%: AKR1B1(−)/AKR1B10(−) plus MRP1(−), 5-year OS, 68%; Intermediate-2 prognosis group, *n* = 6.3%: AKR1B1(−)/AKR1B10(−) plus MRP1(+), median survival, 17 months, 5-year OS, 20%; and Poor prognosis group, *n* = 3, 16%: AKR1B1(−)/AKR1B10(+), median survival, 18 months, 5-year OS, 0%. *n* = 2. **Conclusions**: The Urayasu classification for ALL is considered reliable for predicting the prognosis of patients with ALL after the initial Hyper CVAD/MA remission induction therapy.

## 1. Introduction

At least eight mechanisms of resistance to hematopoietic malignancies have been reported [1]. (1) Activation of drug efflux mechanisms; (2) Suppression of drug influx mechanisms; (3) Activation of drug detoxification pathways; (4) Tumor microenvironment, including immune system evasion; (5) Cancer stem cells; (6) Changes in signal transduction pathways; (7) Gene mutations before and after treatment; and (8) Epigenetic changes. Topics (5) to (8) have been widely studied. Topics (1) to (4) have not received much clinical research, so we have summarized them in references [2,3,4]. We have proposed the Urayasu Prognostic Classification (UC) based on the expression of intrinsic drug resistance proteins for LBCL [2], aggressive T-cell lymphoma (TCL) [3], and AML [4]. The purpose of this study is to propose UC based on the expression pattern of intrinsic drug resistance proteins in de novo ALL. According to the prognostic index of the international ALL trial MRC UKALL XII/ECOG E2993 [5], the Philadelphia chromosome (Ph) indicates a poor prognosis. Poor prognostic factors for a negative Ph are associated with age over 35 years, elevated white blood cell count, and failure to achieve complete remission within 4 weeks. Representative intrinsic drug resistance proteins are described below.

1-1. Microenvironmental Factors

1-1-1. Non-immune microenvironmental factors: Factors that promote overcoming stress conditions such as hypoxia and hypoglycemia in the tumor microenvironment.

1-1-1-1. Glucose-regulated protein 94 (GRP94) [6,7] is primarily found in the endoplasm mic reticulum and mitochondria and is secreted extracellularly to regulate apoptosis, inflammation, and angiogenesis [6]. Therapeutic drugs selectively targeting GRP94 are being developed for cancer [7].

1-1-1-2. Glucose-regulated protein 78 (GRP78) [8,9,10] is primarily expressed in the endoplasmic reticulum and is associated with cancer and is a therapeutic target [8]. It has been identified in high-risk B-ALL patients [9]. Combination therapy with GRP78, CAR-T cells, and dasatinib significantly enhances its effector function [10].

1-1-1-3. Transforming growth factor β1 (TGFβ1) [11,12] is involved in cell proliferation and differentiation, apoptosis, angiogenesis, and cell-mediated immunity.

1-1-1-4. Tumor necrosis factor α1 (TNFα1) [13] is associated with a poorer survival rate in AML patients [13]. It has been reported that fibrosis caused by TGF-β1 and TNFα1 is associated with a poor prognosis [14]. Soluble TNF initiates TNFR1 signaling, but not TNFR2 [15]. TNFR1 promotes AML proliferation [16]. In ALL, TNFR1 and caspase-10 induce cell death [17].

1-1-2. Immune Microenvironment Factors (3 Types)

1-1-2-1 Programmed Cell Death-1 (PD-1) (CD279) [18].

1-1-2-2 Programmed Cell Death Ligand 1 (PD-L1, CD274) [19]. PD-L1 expression is associated with a poor prognosis in ALL.

1-1-2-3 Programmed Cell Death Ligand 2 (PD-L2) [20]. PD-1/PD-L1, 2 expression on the surface of AML tumor cells is important. Patients benefit from immune checkpoint inhibitor therapy.

1-2. Decreased Drug Uptake Activity: Equilibrative nucleoside transporter 1 (ENT1) promotes drug uptake [21]. In T-ALL patients, decreased ENT1 expression in tumors reduces cytarabine influx and may lead to treatment resistance.

1-3. Enhanced Drug Excretion Activity

1-3-1. Multidrug Resistance 1 (MDR1) [22]: MDR1 is a molecular marker for predicting the prognosis of ALL patients.

1-3-2. Multidrug Resistance-Associated Protein 1 (MRP1) [23]: MRP1 expression has a significant impact on the survival of ALL patients.

1-3-3. Multidrug Resistance-Associated Protein 4 (MRP4) [24]: MRP4 suppresses tumor growth and may be a promising target for novel drug therapies.

1-4. Altered Drug Metabolism Activity

1-4-1. Cytochrome P450 3A4 (CYP3A4) [25]: CYP3A4 is involved in the metabolism of many therapeutic drugs.

1-4-2. CYP2B6 [26]: CYP2B6 mutations are associated with the risk of AML.

1-4-3. Aldo-keto reductase family 1 member C3 (AKR1C3) [27]: In acute leukemia, intracellular AKR1C3 degrades doxorubicin, leading to treatment resistance.

1-4-4. AKR1B1 [28]: AKR1B1 is structurally very similar to AR1B10, and the two competitively inhibit each other.

1-4-5. AKR1B10 [29]: The intracellular concentration of daunomycin in tumor cells is primarily reduced by the presence of AKR1C3, and the intracellular concentration of idarubicin is also reduced, although by only about one-fifth the amount. AKR1B10 reduces the intracellular concentrations of not only daunomycin but also idarubicin. Like AKR1C3, it is associated with cisplatin resistance, cyclophosphamide resistance, methotrexate resistance, and vincristine resistance. AKR1B10 expression is controlled by a gene on chromosome 7q33. Chromosome 7 deletions regulate AKR1B10 expression and enhance its function. The Bcr-Abl tyrosine kinase inhibitor dasatinib regulates AKR1B10 expression and inhibits the metabolism of daunomycin and idarubicin, making it a promising treatment for AML [30]. The tyrosine kinase inhibitor ibrutinib regulates AKR1C3 expression and inhibits the metabolism of doxorubicin, making it a promising treatment for AML.

1-5. Other Functional Proteins

1-5-1. Thymidine phosphorylase (TP) [31]: Expression of TP contributes to tumor cell resistance to malnutrition, angiogenesis, invasion, and metastasis, leading to poor prognosis.

1-5-2. P53 [32]: To establish the clinical significance of TP53-mutated ALL, more rigorous characterization of its resistance profile and response to treatment is required.

1-5-3. MYC [33]: An essential ERG- and c-MYC-dependent transcriptional network involved in the regulation of metabolic and ribosome biosynthesis pathways in BCR::ABL1 B-ALL. This network may reveal previously unidentified vulnerabilities and therapeutic targets.

1-5-4. Glutathione sulfate transferase (GST) [34]: The GST1 gene is useful for selecting chemotherapy regimens for AML [35].

## 2. Materials and Methods

The flowchart for the method is as follows: 40 thin, serial sections are prepared as unstained specimens from paraffin blocks used to preserve bone marrow pathology biopsy specimens taken at the time of diagnosis from ALL patients who underwent Hyper CVAD therapy at our hospital. HE staining is performed on the unstained specimens, and immunohistochemistry specimens are prepared using each antibody. Two pathologists examine the specimens microscopically to determine whether each antibody is positive (over 50%) or negative (under 50%). If the determinations differ, a decision is reached through discussion. The statistical correlation between the positivity of each antibody and the survival time of each case is determined using the Kaplan–Meier method.

### 2.1. Patients and Sample Collection

This study enrolled 19 ALL patients who received standard Hyper CVAD/MA combination therapy as initial induction therapy at our hospital between 2015 and 2020. The distribution of patient disease types is shown in Table 1. Pretreatment paraffin-embedded biopsy specimens from patients were subjected to immunohistochemistry (IHC) to examine the expression of 23 proteins previously reported as treatment resistance factors. Positive and negative staining was determined by light microscopy. Data were collated and analyzed retrospectively. Analytical models included variables such as anticancer drug metabolism factors, and overall survival (OS) after induction therapy was compared using the log-rank test. The MRC UKALL XII/ECOG classification [5] was used for comparison. The approval code for this study by our hospital’s ethics committee was U17-0016, and it was approved on 9 December 2022.

### 2.2. IHC

IHC was performed. Primary antibodies against key proteins involved in anticancer drug metabolism were used: (1) GRP94: Proteintech (Rosemont, IL, USA), clone 1H10B7 (monoclonal antibody); (2) CYP3A4: Sigma-Aldrich (St. Louis, MO, USA), SAB1400064 (polyclonal antibody against CYP3A4); (3) AKR1C3: Proteintech 11194-1-AP (polyclonal antibody against AKR1C3); (4) MDR1 (P-glycoprotein): Proteintech, 22336-1-AP (polyclonal antibody against MDR1); (5) MRP1 (CD9): Proteintech, 60232-1-IG (monoclonal antibody against full-length MRP1); (6) TGF-beta1: Proteintech, 21898-1-AP (polyclonal antibody against TGF-beta); (7) GRP78: Proteintech, 66574-1-IG (monoclonal antibody against full-length GRP78); (8) Glutathione S-transferase-κ1 (GST): Proteintech, 14535-1-AP (polyclonal antibody against GST1); (9) Thymidine phosphorylase: Abcam (Cambridge, UK), ab226917 (polyclonal antibody); (10) MRP4 (ABCC4): SANTA CRUZ BIOTECHNOLOGY (Dallas, TX, USA), SC-376262 (monoclonal antibody against MRP4); (11) CYP2B6: LifeSpan BioSciences, Inc. (Seattle, WA, USA), LS-C352084 (polyclonal antibody against CYP2B6); (12) TNF1-alpha: Sigma-Aldrich, SAB4502982 (polyclonal antibody against TNF1-alpha) (13) PD-1; (14) PD-L1: Proteintech, 66248-1-IG, monoclonal antibody, clone 2B11D11; (15) PD-L2: Proteintech, 18251-1-AP16, rabbit IgG polyclonal antibody; (16) P53: Cell Signaling Technology, Inc. (3 Trask Lane, Danvers, MA, USA), DO-7 mouse monoclonal antibody #48818; (17) c-MYC: Abcam (Kendall Square, Cambridge, MA, USA), Y69 clone ab32072; (18) ENT-1: Proteintech, 1337-1-AP, IgG polyclonal antibody; (19) AKR1B1: Sigma-Aldrich (3050 Spruce Street, Saint Louis, MO, USA), polyclonal antibody HPA052751; and (20) AKR1B10: Sigma-Aldrich (3050 Spruce Street, Saint Louis, MO, USA), monoclonal antibody HPA020280. After immunostaining, two pathologists definitively interpreted the IHC staining results. IHC staining was considered positive if 50% or more of the tumor cells showed positive staining. The agreement rate between the two pathologists was approximately 81%. In cases of disagreement, the final diagnosis was made by consensus.

Polyclonal antibodies bind to multiple epitopes, which means they are more prone to inherent variability than monoclonal antibodies. However, by taking the following measures, we can significantly improve the consistency of immunohistochemistry (IHC) results: standardizing and strictly adhering to protocols, quality control of antibodies and addressing batch-to-batch differences, thorough controls, and using automated stainers. By combining these measures, we can significantly improve the consistency of IHC results, even when using polyclonal antibodies.

Optimizing IHC markers involves appropriately setting the following experimental conditions to specifically and efficiently detect the target antigen. Please contact us and we will provide you with detailed information on antigen retrieval methods, primary antibody dilution ratios, and blocking conditions. Positive controls can also be provided.

### 2.3. Statistical Analysis

To confirm the association between the OS and poor prognostic factors/factors involved in anticancer drug metabolism after the initial Hyper CVAD/MA therapy, survival curves were plotted using the Kaplan–Meier method, and factors significantly associated with the OS were evaluated using the log-rank test. The significance level in the statistical tests was set at 0.05 (two-tailed), and *p* < 0.05 indicated a statistically significant difference. Statistical analyses were performed using EZR version 2.7-1 software (Saitama Medical Center, Jichi Medical University, Saitama, Japan) [36].

If the sample size is too small, even if the log-rank test produces a “significant” result (usually a *p*-value of less than 0.05), the results may be unreliable. As a countermeasure, this study clarified that more data is needed to draw conclusions. We focused on presenting survival curves and visually illustrating the trends in the data as case report article.

Multiple comparisons were not considered because of the exploratory nature of this study.

## 3. Results

Table 1 compares the prognostic factor classification stratification study of the international ALL trial: MRC UKALL XII/ECOG E2993, published in Blood in 2005 [5]. Table 1 shows no significant difference in 5-year survival rate between the two. The proportion of Philadelphia chromosome-positive patients in this study was significantly higher than in the MRC UKALL study.

### 3.1. Comparison of Survival Times Between Groups Using Kaplan–Meier Survival Curves and the Log-Rank Test

**Figure 1:**
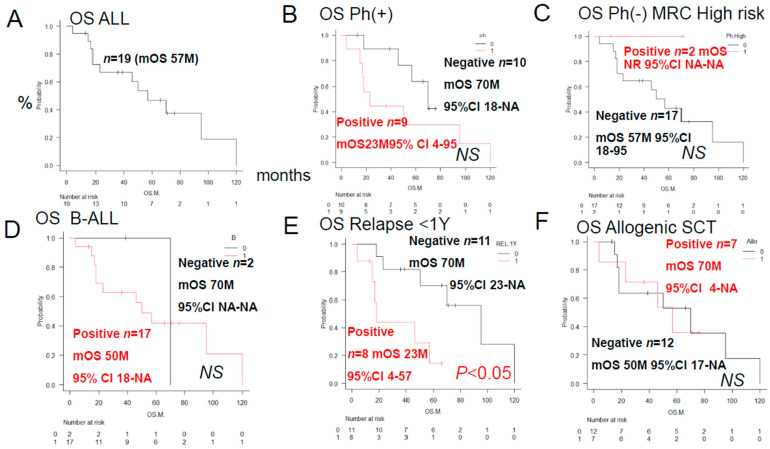
Overall survival rates of ALL patients by prognostic factors after initial induction therapy with Hyper CVAD/MA. Kaplan–Meier (KM) survival curves and comparisons of disease and prognostic factors between the two groups were performed using the log-rank test (red, black) 95% confidence intervals (CI) have been added to all KM graphs. (**A**) Overall survival (OS) for all patients: median OS was 57 months, and the 5-year survival rate was 48%. (**B**) There was no significant difference between the Philadelphia chromosome (Ph)-positive and -negative groups (not significant [NS]). (**C**) There was no significant difference in OS between the Ph-negative, MRC high-risk positive, and MRC high-risk negative groups. (**D**) There was no significant difference in OS between patients with B-ALL (red) and T-ALL (black). (**E**) OS and 5-year survival rates were significantly reduced in patients who relapsed within 1 year of treatment (*p* < 0.05). (**F**) There was no significant difference in OS between the group that received and did not receive allogeneic stem cell transplantation.

When comparing survival times in Figure 1, the Philadelphia chromosome-positive group (high-risk group in the MRC UKALL classification) was compared with the negative group (Figure 1B), and the high-risk group within the Philadelphia chromosome-negative group was compared with the other groups (Figure 1C). In other words, the ALL cases in this study were not well classified using the MRC UKALL classification.

#### 3.1.1. Overall Survival Time of ALL Patients (Various Prognostic Factors)

The median overall survival time for 19 patients was 57 months after initial induction therapy, and the 5-year overall survival rate was 44% (Figure 1). Overall survival time was compared using the log-rank test in relation to whether tumor cells were positive/negative for anticancer drug metabolic factors. There were no significant differences in the OS rates among the three patient groups classified according to the ALL MRC UKALL classification or between the groups that did and did not undergo allogeneic stem cell transplantation. However, a significant difference in the relapse rate within one year was seen between the latter two groups of patients.

#### 3.1.2. Overall Survival (OS) of ALL Patients According to the Presence or Absence of Prognostic Factor Expression After Immunohistochemical Staining (IHC)

**Figure 2:**
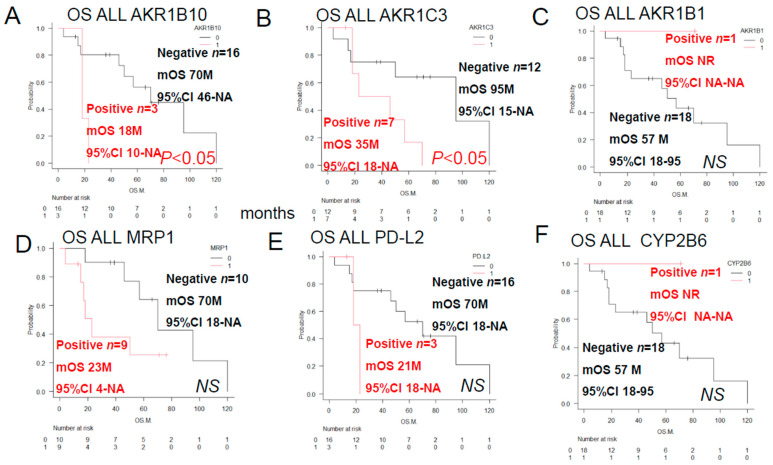
Overall survival rate of ALL patients with and without prognostic factors. Kaplan–Meier (KM)survival curves and prognostic factors were compared between groups using the log-rank test (red indicates immunostaining-positive groups, black indicates immunostaining-negative groups. 95% confidence intervals (CI) have been added to all KM graphs. (**A**) OS was significantly shortened in the AKR1B10-positive group (*p* < 0.05). (**B**) OS was significantly shortened in the AKR1C3-positive group (*p* < 0.05). (**C**) There was no significant difference in OS between the AKR1B1-positive and -negative groups (NS). (**D**) There was no significant difference in OS rate between the MRP1-positive and -negative groups. (**E**) There was no significant difference in OS between the PD-L2-positive and -negative groups. (**F**) There was no significant difference in OS between the CYP2B6-positive and -negative groups. It is known that the log-rank test, a common test for KM curves, does not allow for reliable statistical conclusions because the sample size is too small. Therefore, we retested Figure 1 and Figure 2 using the Pet-Pet-Wilcoxon method, which is often used for small samples. The results were generally the same, except for MRP1 in Figure 2D, which changed from NS to *p* < 0.05. However, this change did not have much impact on the overall discussion.

As shown in Figure 2, a log-rank test was performed to compare the overall survival (S) of ALL patients classified by the expression of anticancer drug-metabolizing enzymes and pumps. Significant differences were observed in the expression of AKR1B10 and AKR1C3. However, no significant differences in overall survival (OS) were observed between patients expressing AKR1B1, MRP1, PD-L2, or CYP2B6.

#### 3.1.3. Univariate Analysis of Histological Immunostaining Revealed That the Overall Survival Rate of ALL Patients Differed Depending on the Presence or Absence of Each of the Two Prognostic Factors (Figure 3)

The overall survival rate significantly differed between patients with different patterns of the enzymes AKR1C3, AKR1B10, and AKR1B1 (which competitively inhibits AKR1B10), the pump MRP1, and the Philadelphia chromosome. The survival rates also differed significantly between patients with different combinations of AKR1C3 and AKR1B10 expression, AKR1B1 and AKR1B10 expression, AKR1B10 and CYP2B6 expression, MRP1 and AKR1C3 expression, and Philadelphia chromosome status and AKR1C3.

**Figure 3:**
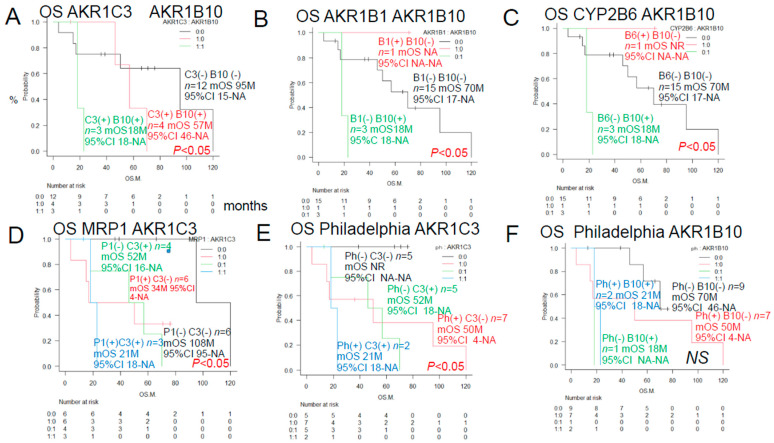
We examined the correlation between AKR1B10, AR1C3, AKR1B1, CYP2B, MRP1, and the expression of the Philadelphia chromosome, AKR1C3, and AKR1B10 and OS of ALL patients. Kaplan–Meier survival curves and each of two prognostic factors were compared between the two groups using the log-rank test. OS is shown in blue if both prognostic factors are positive, black if both are negative, and red or green if one is positive. See Table 2 for AKR1B10 and other factors. 95% confidence intervals (CI) have been added to all KM graphs. Comparing the same data multiple times increases the likelihood of false positive results, so below we used the EZR method to adjust for multiplicity using KM’s Bonferroni method to calculate the *p* value. (**A**) AKR1B10 and AKR1C3 showed a significant difference in OS (*p* < 0.05). (**B**) AKR1B1 and AKR1B10 showed a significant difference in OS (*p* < 0.05). (**C**) MRP1 and AKR1C3 showed a significant difference in OS (*p* < 0.05). (**D**) MRP1 and AKR1C3 showed a significant difference in OS (*p* < 0.05). (**E**) Philadelphia chromosome (Ph) and AKR1C3 showed a significant difference in OS (*p* < 0.05). (**F**) Ph and AKR1B10 showed no significant difference in OS.

**Table 2:** OS was significantly different between patients with different expressions of prognostic factors (results of univariate analysis).

Category	Subcategory	Factors (♯Significant Difference:)	*n*	Median OS (Months)	Years Survival Rate	*p* Value	Figure
**Total**	**ALL**	**All Patients**	**19**	**57M**	**5Y 44%**		**Figure 1A**
**ELN**	**MRC classification for ALL**	**MRC ph (+)**	**9**	**23M**	**5Y 28%**	**NS**	**Figure 1B**
		**MRC ph (−) High risk**	**2**	**NR**	**5Y100%**	**NS**	**Figure 1C**
		**MRC ph (−) Standard risk**	**8**	**70M**	**5Y 58%**	**NS**	**Figure 1D**
**Other prognostic factor**	**Within 1Y relapse**		**8**	**23M**	**5Y 14%**	* *p* **< 0.05**	**Figure 1E**
	**Allogenic transplantation**		**7**	**70M**	**5Y 36%**	**NS**	**Figure 1F**
**CHO metabolic enzyme**	**AKR1B1 family**	**AKR1B10 (** **♯** **)**	**3**	**18M**	**5Y 0%**	* *p* **< 0.05**	**Figure 2A**
		**AKR1C3**	**7**	**35M**	**5Y 18%**	* *p* **< 0.05**	**Figure 2B**
		**AKR1B1**	**1**	**NR**	**5Y100%**	**NS**	**Figure 2C**
	**Fibrosis**	**Silver stain**	**6**	**95M**	**5Y 78%**	**NS**	
	**HO efflux pump**	**MDR1**	**0**				
		**MRP1**	**9**	**23M**	**5Y 22%**	**NS**	**Figure 2D**
**MTX efflux pump**	**MTX efflux pump**	**MRP4**	**0**				
**Immune check point**	**Immune check point**	**PD-1**	**0**				
		**PD-L1**	**0**				
		**PD-L2**	**3**	**21M**	**5Y 0%**	**NS**	**Figure 2E**
**OH metabolic enzyme**	**C activating enzyme**	**CYP2B6**	**1**	**NR**	**5Y 100%**	**NS**	**Figure 2F**
	**CHOP metabolic enzyme**	**CYP3A4**	**0**				
**Microenvironment**	**ER stress proteins**	**GRP78**	**8**	**70M**	**5Y 56%**	**NS**	
		**GRP94**	**13**	**57M**	**5Y 48%**	**NS**	
		**TGF beta1**	**7**	**50M**	**5Y 42%**	**NS**	
		**TNF alpha1**					
**Others**		**GST**	**7**	**73M**	**5Y 50%**	**NS**	
		**Ki-67**	**12**	**46M**	**5Y 22%**	**NS**	
		**MYC**	**5**	**40M**	**5Y 0%**	**NS**	
		**P53**	**1**	**NR**	**5Y 100%**	**NS**	
		**TP**	**0**				
**Significant combination**	**AKR1 family**	**AKR1C3(+), AKR1B10(+) (** **♯** **)**	**3**	**18M**	**5Y 0%**	* *p* **< 0.05**	**Figure 3A**
**other than Urayasu**		**AKR1B1(−), AKR1B10(+) (** **♯** **)**	**3**	**18M**	**5Y 0%**	* *p* **< 0.05**	**Figure 3B**
**classification for ALL**		**CYP2B6(−), AKR1B10(+) (** **♯** **)**	**3**	**18M**	**5Y 0%**	* *p* **< 0.05**	**Figure 3C**
		**MRP1(+), AKR1C3(+) (** **♯** **)**	**3**	**21M**	**5Y 0%**	* *p* **< 0.05**	**Figure 3D**
		**Ph(+), AKR1C3(+) (** **♯** **)**	**2**	**21M**	**5Y 0%**	* *p* **< 0.05**	**Figure 3E**
		**Ph(+), AKR1B10(+)**	**2**	**21M**	**5Y 0%**	**NS**	**Figure 3F**
**Urayasu classification**	**Group 1**	**AKR1B1(+), 1B10(−) (** **♯** **)**	**1**	**NR**	**5Y 100%**	* *p* **< 0.05**	**Figure 3B and Figure 4A**
**for ALL**	**Group 2**	**AKR1B1(−), 1B10(−)MRP1(−) (** **♯** **)**	**9**	**70M**	**5Y 48%**	* *p* **< 0.05**	**Figure 4B**
	**Group 3**	**AKR1B1(−), 1B10(−)MRP1(+) (** **♯** **)**	**6**	**17M**	**5Y 20%**	* *p* **< 0.05**	**Figure 4B**
	**Group 4**	**AKR1B1(−), 1B10(+) (** **♯** **)**	**3**	**18M**	**5Y 0%**	* *p* **< 0.05**	**Figure 3B and Figure 4A**

Note: (♯) or * *p* < 0.05 indicates statistically significant difference at *p* < 0.05.

### 3.2. Urayasu Classification for ALL

**Figure 4:**
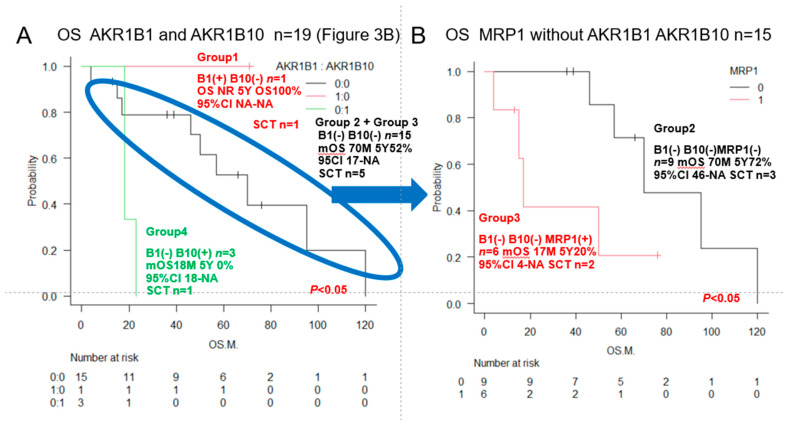
Urayasu classification for ALL (groups 1–4). Group 1 (Good prognosis group): AKR1B1 (+) AKR1B10(−), *n* = 1, median OS not reached, 5-year OS rate 100%. Group 2 + Group 3 (Intermediate prognosis): AKR1B1 (−) AKR1B10(−), *n* = 15, median OS 70 months, 5-year OS rate 52%. Group 4 (Poor prognosis): AKR1B1 (−) AKR1B10(+), *n* = 3, median OS, 18 months, 2-year OS rate 0%. Group 2 (Intermediate-1 prognosis): AKR1B1 (−) AKR1B10(−) MRP1(−), *n* = 9, median OS 70 months, 5-year OS rate 72%. Group 3 (Intermediate-2 prognosis): AKR1B1 (−) AKR1B10(−) MRP1(+) *n* = 6, median OS 17 months, 5-year OS rate 20%.

As shown in Figure 4, we propose the Urayasu classification of ALL based on the expression of not only MRP1 but also treatment resistance factors (AKR1B1 and AKR1B10), as follows:

- Group 1 (good prognosis group): AKR1B1(+) AKR1B10 (−), *n* = 1.
- Group 2: AKR1B1(−) AKR1B10(−) MRP(-), *n* = 9.
- Group 3: AKR1B1(−) AKR1B10(−) MRP(+), *n* = 6.
- (Group 2 and Group 3 showed a more favorable prognosis than Group 4.)
- Group 4 (poor prognosis group): AKR1B1(−) AKR1B10(+), *n* = 3. Significant differences in the OS rates were observed among the four groups. These results are also shown in Table 2.

Although the number of cases was small and this classification is only for reference, the Urayasu Classification in Figure 4 showed significant differences in survival time among the four groups, making it possible to stratify patients. This is very interesting.

Table 2 shows the results of immunohistochemical analysis for 23 treatment resistance factors. The median survival rate and 95% confidence interval (CI) of ALL19 patients calculated by the Kaplan–Meier (KM) method, as well as intergroup comparison (*p*-value: log rank test) are shown. Poor prognostic factors were evaluated based on the difference in survival time, and significant differences (*p* < 0.05) are marked with a #. The outcomes were compared with those in the three groups of patients classified according to the MRC classification, which is the conventional prognostic classification.

### 3.3. Case A Presentation: Urayasu Classification for ALL (Figure 5)

**Figure 5:**
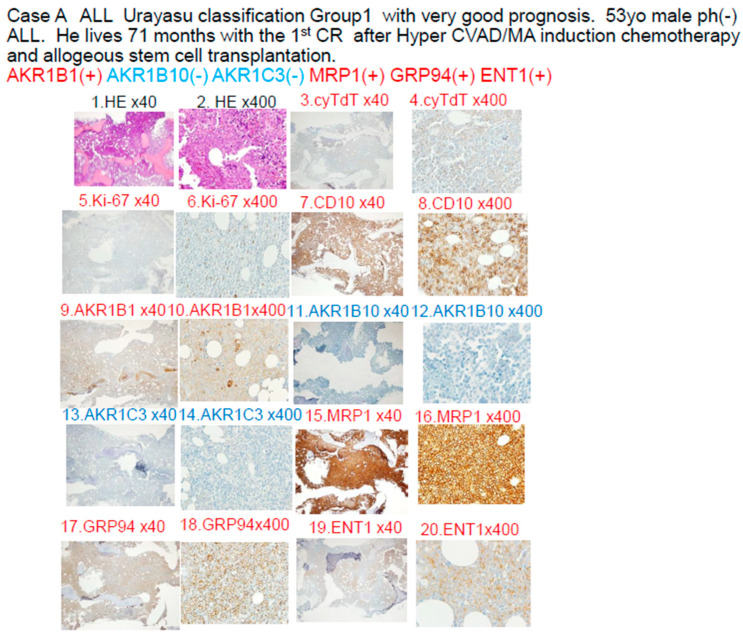
Case A: A 52-year-old male. In April 2019, he visited our hospital complaining of testicular swelling, oral bleeding, and bloody stools. His white blood cell count was 100,000/μL, his LDH was 2000 IU, and his platelet count was 8000/μL. His bone marrow biopsy revealed Philadelphia chromosome-negative B-ALL. He achieved complete remission with Hyper CVAD therapy. He underwent three cycles of MA consolidation therapy and alternating Hyper CVAD/MA therapy. He underwent allogeneic hematopoietic stem cell transplantation in October 2019 and has survived 71 months without recurrence. At 400× magnification, both TdT and Ki-67 were weakly positive.

Figure 5 Low- and high-power light microscopic pathological findings after immunohistochemical staining of a biopsy specimen (lymph node) from a Case A UG1 case.

### 3.4. Abstract Schema in This Study

**Figure 6:**
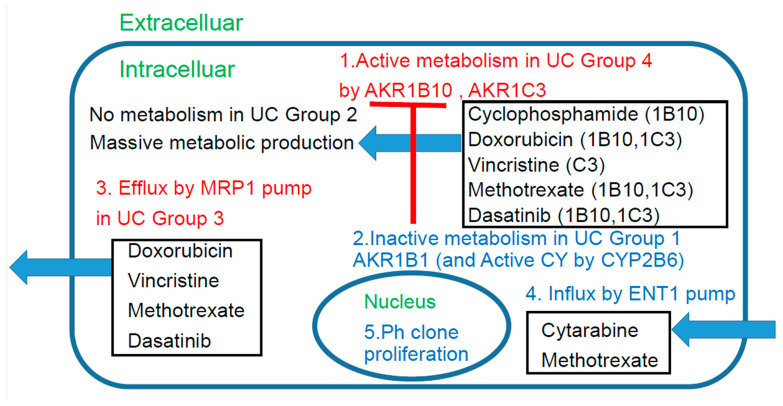
Chemoresistance mechanism in ALL cells.

Abstract schema of this study is shown in Figure 6.

Future AKR1B10 inhibitor therapy may require individualized treatment. AKR1B10 shares 70.6% amino acid sequence identity with the aldose reductase enzyme AKR1B1, and their structures and substrate specificities are very similar. Many compounds that inhibit AKR1B10 also inhibit AKR1B1 to the same degree. AKR1B10 positivity also confers resistance to cisplatin. It is also known to confer a high degree of resistance to cyclophosphamide (CY), which may reduce the effectiveness of CY used in allogeneic transplant conditioning regimens such as CY + YBI and CY + busulfan, potentially increasing the relapse rate. AKR1B10 expression is regulated by a gene on chromosome 7q33. Chromosome 7 deletions may result in activation of AKR1B10. The Bcr-Abl tyrosine kinase inhibitor dasatinib inhibits AKR1B10 and inhibits the metabolism of daunomycin and idarubicin, making it promising for therapeutic application. NSAIDs such as N-phenyl-anthranilic acid, diclofenac, and glycyrrhetinic acid competitively inhibit AKR1B10. Based on the above, dasatinib and other NSAIDs are considered promising AKR1B10 inhibitors for stratified treatment of ALL.

### 3.5. Summarization of the Review of the Literature [2,3,4] Plus Findings of This Study (Figure 7)

This is a summary of the Urayasu classification based on treatment resistance factors for four aggressive hematopoietic tumors.

Regarding the relationship between treatment resistance factors in the Urayasu classification and this study’s LBCL (L) [2], TCL (T) [3], AML (AM) [4], and ALL (AL) [this study], this is a summary of the Urayasu classification based on treatment resistance factors for four aggressive hematopoietic tumors. The prognostic significances of the treatment resistance factors in the Urayasu classification for LBCL (2), UC for TCL (3), UC for AML (4), and UC for ALL (current study) are summarized in a Venn diagram for each disease. There were no factors that were common to all four diseases. Common factors in the first three diseases were the growth factor P53 (LBCL 36%, TCL 13%, AML 9%), which was more prevalent in LBCL, the efflux pump MRP1 (LBCL 25%, AML 9%, ALL 46%), and AKR1C3, the enzyme that metabolizes doxorubicin, methotrexate, vincristine, and dasatinib (LBCL, TCL, ALL). Common to LBCL, TCL were the microenvironmental adaptation factor, ER stress protein GRP94, and common to AML and ALL was AKR1B10, which metabolizes idarubicin, daunorubicin, cyclophosphamide, and cisplatin. Dasatinib inhibits AKR1B10. The tyrosine kinase inhibitor ibrutinib inhibits AKR1C3, thereby inhibiting doxorubicin metabolism and showing promise for potential therapeutic application. Five survival activators (PD-1, PD-L1, TP, GRP78, and GRP94), which are microenvironmental factors, are also important in TCL. CYP3A4, which detoxifies doxorubicin, and the doxorubicin efflux pump MDR1 are important in LBCL. The HO efflux pump MRP1 is more frequently expressed in ALL, AML, and LBCL than in TCL, which is dependent on microenvironmental adaptation factors. P53 is involved in many diseases other than ALL. However, the poor prognosis of TCL is largely due to p53 expression. p53 is an important tumor suppressor, and loss of p53 function due to mutations or other factors leads to cancer development. p53 mutations occur in more than 50% of human cancers. However, currently, no drugs have been approved for the clinical treatment of cancers expressing mutant p53. LBCL, AML, and TCL are most frequently positive for AKR1C3, an enzyme that inactivates doxorubicin, cyclophosphamide, and vincristine (AML 17/35: 46%, TCL 6/16: 38%, LBCL 26/42: 62%). Based on these findings, while R-CHOP and Pola-RCHP regimens are standard therapies, many LBCL tumors express AKR1C3, which may contribute to treatment resistance. AML patients with P53 (3/35 9%) or MRP1 (1/35 3%) mutations show a poor prognosis. Although the incidence rate is low (12%), even with combined treatment, MRP1 inhibitors have been developed. In addition to significant microenvironmental factors in TCL, AML cells can express AKR1B10, a drug that metabolizes the anthracycline anticancer drug idarubicin, which can result in treatment resistance. AKR1B1 shares 70.6% amino acid sequence identity with AKR1B10 and shares highly similar structure and substrate specificity. Many compounds that inhibit AKR1B10 also competitively inhibit AKR1B1 to a similar degree, reducing AKR1B10 activity and reversing treatment resistance. The Abl tyrosine kinase inhibitor dasatinib inhibits AKR1B10, inhibiting daunomycin and idarubicin metabolism, offering promise for potential therapeutic application. AKR1B10 is believed to be the primary pathway underlying cisplatin and cyclophosphamide resistance [29]. In fact, caution is required in the case of AKR1B10-positive ALL, in which high-dose cyclophosphamide therapy used as a conditioning regimen for allogeneic hematopoietic stem cell transplantation may show reduced efficacy. Furthermore, AKR1B10 is regulated by a gene on chromosome 7q33. Loss of chromosome 7 may result in the activation of AKR1B10. Meanwhile, TCL has a superior ability to adapt to the microenvironment, and LBCL is also thought to have a superior ability to detoxify anticancer drugs.

**Figure 7:**
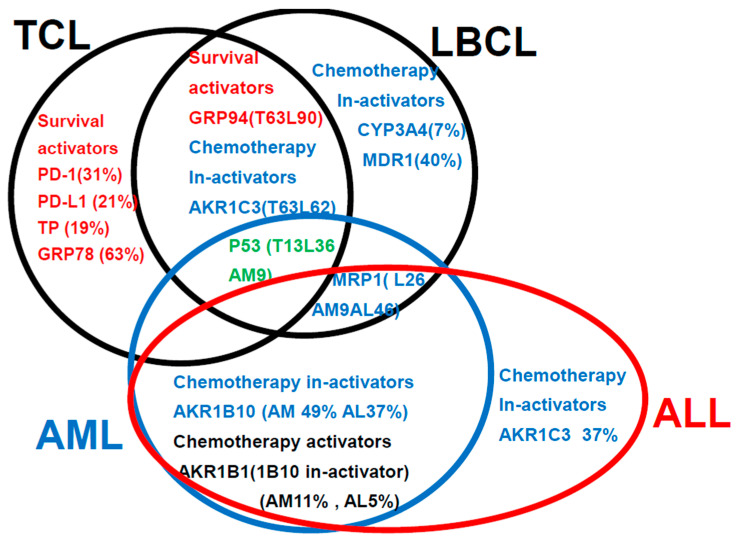
The review of the literature [2,3,4] plus findings of this study.

## 4. Discussion

Among the mechanisms of resistance to anticancer drugs in hematopoietic malignancies, those involving a decrease in intracellular anticancer drug concentrations appear to be especially important, although there have been no comprehensive investigations of their association with clinical treatment resistance. In this study, we focused on the following mechanisms that can reduce the intracellular drug concentrations of anticancer drugs: (1) enhanced activities of anticancer drug efflux mechanisms; (2) suppressed activities of drug influx mechanisms; (3) enhanced activities of drug detoxification pathways; and (4) influence of the tumor microenvironment. In Section 4.1, Section 4.2 and Section 4.3, we describe our study involving 19 cases of ALL (42 cases of LBCL in [2], 16 cases of TCL in [3], and 35 cases of AML in [4]) that underwent initial induction therapy at our hospital; we performed immunohistochemical analysis for 23 resistance-associated proteins in tumor specimens obtained from these patients. We retrospectively analyzed the correlations between the OS duration/rate of the patients and results of the immunohistochemical analyses by the Kaplan–Meier method. In Section 4.4, we summarize the results of a literature search conducted by us to review the proposed drug resistance mechanisms in hematopoietic malignancies. Based on the clinical study and review of the literature, we have comprehensively demonstrated associations between the mechanisms of treatment resistance involving a decrease in intracellular anticancer drug concentrations and clinical treatment resistance.

### 4.1. Discussion Based on the Urayasu Classification for ALL Proposed in This Study (Table 1 and Table 2, Figure 1, Figure 2, Figure 3 and Figure 4)

In general, immunohistochemistry is considered an extremely useful tool as it allows for determinations of positive/negative results based on the observation of tumor cells under an optical microscope. Positivity is defined as positive staining, including weakly positive staining, in ≥50% of tumor cells. In this study of ALL patients, we performed immunohistochemical analyses in tumor specimens obtained from 19 cases of ALL for 23 treatment resistance factors (6–34) determined as being clinically important based on a review of the literature (Table 1). In this cohort of ALL patients, there were no significant differences in the OS among patient groups classified according to the MRC UK ALLXIIECOG E2993 classification (Figure 1B–D). It is known that the log-rank test, a common test for KM curves, does not allow for reliable statistical conclusions because the sample size is too small. Therefore, we retested Figure 1 and Figure 2 using the Pet-Pet-Wilcoxon method, which is often used for small samples. The results were generally the same, except for MRP1 in Figure 2D, which changed from NS to *p* < 0.05. However, this change did not have much impact on the overall discussion. However, as shown in Figure 4A,B, the Urayasu classification for ALL allowed reliable prognostic stratification of the patients, with significant differences in the OS observed among the patients classified into the four groups according to this classification. Therefore, chemotherapy resistance factors are more specific for prognostic classification of ALL. We propose the Urayasu classification (UC) for ALL as a useful classification for predicting the outcomes after Hyper CVAD/MA therapy in newly diagnosed ALL patients (Figure 4A,B). The UC for ALL (groups 1 to 4) is shown below. In this study, the overall 5-year OS rate of the patients (*n* = 19) was approximately 43%. The 5-year OS rate of patients (*n* = 1521) classified by the MRC UKALLXIIECOG E2993 classification was approximately 38.5% (5). The 5-year OS rate was 100% in UC Group 1 (favorable prognosis group; AKR1B10(−) AKR1B1(+); *n* = 1) versus 57% in the MRC UKALLXIIECOG E2993- Ph-negative Standard group. The 5- year OS was 48% in UC Group 2 (intermediate-1 prognosis group; AKR1B10(−) AKR1B1(−) MRP1(−); *n* = 9) versus 35% in the MRC UKALLXIIECOG E2993-Ph-negative High group (*n* = 594). The 5-year OS was 20% in UC Group 3 (intermediate-2; AKR1B10(−) AKR1B1(−) MRP1(+); *n* = 6). The 5-year OS rate was 0% in Group 4 (poor prognosis group; AKR1B10(+) AKR1B1(−); *n* = 3). As seen from the above, although the number of cases was small, the UC for ALL allows more reliable prognostic classification of patients into good- and poor-prognosis groups as compared with the UKALLXIIECOG E2993 classification (Figure 1B,C). In Figure 4A,B, the breakdown of the number of patients who underwent stem cell transplantation in each prognostic group is listed after SCT (stem cell transplantation). The number of patients was almost evenly distributed across all prognostic groups. Considering this, along with the fact that there was no significant difference in OS between patients with and without stem cell transplantation (Figure 1F), we believe that stem cell transplantation has little impact on OS. Considering the reasons for this, most of the conditioning treatments for the seven allogeneic stem cell transplants were TBI + CY, and according to the theory in Figure 6, it is easy to understand that AKR1B10, which reduces CY metabolism, and CYP2B6, which increases CY metabolism (the same example as AKR1B1), are involved. Furthermore, according to reference [37], radiation (TBI) resistance is mediated by AKR1B10. In addition, the initial induction therapy for all patients was the same HyperCVAD/MA treatment, and according to the theory in Figure 6, the metabolism of AKR1B10, AKR1B1 (CYP2B6), and MRP1 is most important, which seems to be consistent with the results of this study. We believe that this classification could contribute to stratification of treatment and promote development of treatment methods based on the mechanisms of treatment resistance.

### 4.2. Discussion Based on the Study of ALL Cases (Figure 5)

In Case A, the blast cells at the time of diagnosis showed positive IHC staining for the factors known to enhance the therapeutic efficacy AKR1B1 and ENT1. The therapeutic inhibitors were negative for AKR1B10, AKR1C3, and CYP3A4, but positive staining for MRP1. The tumor was negative for AKR1B10 and AKR1C3, and the staining result was negative for enzyme AKR1. The patient was classified into Group 1 of the UC for ALL. The tumor was positive for the metabolic enzyme AKR1B1, suppressed for AKR1B10, and negative for AKR1B10 and AKR1C3. Thus, dasatinib, cyclophosphamide, doxorubicin, vincristine, and methotrexate were expected to be effective, and proved to be effective even in the presence of expression of the efflux pump MRP1. Cytarabine and methotrexate may have been highly effective as the positive tumor cell expression of the influx pump ENT1 would have allowed large amounts of these drugs to be taken up by the tumor cells. It is believed that complete remission was achieved quickly after Hyper CVAD/MA therapy and maintained for a long period of time based on the above pattern of expression of the prognostic factors.

### 4.3. Discussion Based on the Study of ALL Cases (Figure 6)

Figure 6 discusses the effects of the expression patterns of the enzymes in Groups 1–4 of the UC for ALL described in Figure 4. For LBCL, TCL, and ALL, for which CHOP-like regimens are often selected as the initial regimens, the expression statuses of the HO (hydroxyl doxorubicin, oncovin)-metabolizing enzymes AKR1C3 and AKR1B10 are important. These enzymes are also involved in methotrexate metabolism. AKR1B10 plays a central role in cyclophosphamide metabolism. Furthermore, it is reasonable to assume that dasatinib has an AKR1B10-inhibitory effect in Ph-positive ALL cases. All drugs but cyclophosphamide are excreted by the MRP1 pump. Cytarabine enters the cells via the ENT-1 pump.

### 4.4. Discussion Based on the Findings of Both Our Previous Studies [1,2,3] and This Study (Figure 7)

Figure 7 summarizes the prognostic factors for aggressive hematopoietic tumors determined from previous studies [2,3,4] and this present study of ALL. Treatment resistance factors include microenvironmental factors and factors reducing the tumor cell concentrations of anticancer drugs. (1) LBCL: According to reference [1], the median survival time (OS) was 64 months, and microenvironmental factors included a high expression rate of GRP94 (90%) of factors reducing the intracellular concentrations of anticancer drugs (CYP3A4 7%, AKR1C3 62%, AKR1B10 62%, MDR1 40%, MRP1 26%), and of P53 (36%), all of which are treatment resistance factors. (2) TCL: According to reference [2], the OS was 34 months, representing a poor prognosis. Treatment resistance factors were mainly factors related to the tumor microenvironment (GRP94 63%, GRP78 63%, PD-1 31%, PD-L1 24%, TP 19%). Therefore, even if complete remission is achieved with standard chemotherapy alone, recurrence is likely to develop even if there is minimal residual disease containing cells expressing GRP94. In cases where complete remission was not achieved, AKR1C3 (63%) and P53 (13%) could have been involved. (3) AML: According to reference [3], the 5-year OS was 73%. Treatment resistance factors included factors reducing the tumor cell concentrations of anticancer drugs such as P53 (9%), MRP1 (9%), and AKR1B10 (49%). The microenvironment factor GRP94 was expressed at a high frequency of 94%, but it did not have any significant influence on the treatment efficacy. (4) ALL: According to the results of this study, the OS duration was 57 months, indicative of poor prognosis. Because Hyper CVAD treatment is powerful, many patients achieved temporary complete remission, but many were also at a high risk of relapse. The main treatment resistance factors were factors that reduced the tumor cell concentrations of anticancer drugs, including AKR1B10%, MRP1 46%, and AKR1C3 37%, which increased the risk of relapse. AKR1B1 is a factor that antagonizes AKR1B10 that enhances the efficacy of chemotherapy and is important in ALL (5%). (5) Common treatment resistance factors: There are no common treatment resistance factors across the four diseases. However, the two common treatment resistance factors across the first three diseases are P53 (LBCL 36%, TCL 19%, AML 9%) and MRP1 (ALL 46%, LBCL 26%, AML 9%).

To gain a deeper understanding of resistance to chemotherapy, molecular-targeted drugs and other therapeutic agents are shown in Figure 7.

Based on the results of immunohistochemical analysis, a simple Urayasu 4 classification for ALL has been established for three types of treatment resistance proteins (AKR1B10, AKR1B1, and MRP1). This makes it possible to treat ALL with inhibitors that target the proteins that cause treatment resistance. We hope to verify this by analyzing further cases in the future. Recently, the gut microbiome has been shown to significantly influence the outcome of acute leukemia, especially in patients undergoing hematopoietic stem cell transplantation (HSCT). Disruption of the gut microbiota caused by chemotherapy, antibiotics, and alterations in the immune system contributes to complications such as graft-versus-host disease (GVHD), gastrointestinal disorders, and infections. Further research is needed to optimize microbiome-based therapies and ensure their safety and efficacy in the treatment of acute leukemia. In the future, we would like to investigate other hematological malignancies, elucidate the mechanisms of treatment resistance, and use this information to aid in treatment [37]. We added the following text to the Section 4: Like TARGET, there are many publicly available RNA sequencing data sets with complete cytogenetic and outcome data. Although these data only provide RNA-level expression levels of target genes, they would still serve as a good validation set for validating the model.

## 5. Conclusions

We proposed the Urayasu prognostic classification for ALL. It consists of Group 1 (good prognosis), Group 2 (intermediate prognosis-1), Group 3 (intermediate prognosis-2), and Group 4 (poor prognosis). The use of AKR1B10 and MRP1 inhibitors may improve treatment outcomes in the future.

## Figures and Tables

**Table 1:** Comparison of the WHO classification of this study’s cases for ALL patients, outcomes after Hyper-CVAD/MA induction, whether or not they underwent allogeneic hematopoietic stem cell transplantation, and out-comes accI addedording to the MRC UKAL prognostic classification.

Characteristics of ALL Patients in This Analysis	This Study	MRC UKALL(5)	*p* (*t*-Test)
	*n* = 19	*n* = 995	
WHO Classification, 5th edition			
B-cell lymphoblastic leukaemias/lymphomas	17 (89%)	627 (63%)	
B-lymphoblastic leukaemia/lymphoma with BCR-ABL1 fusion	10 (53%)	239 (24%)	*p* < 0.05
B-lymphoblastic leukaemia/lymphoma, NOS	4 (21%)	nd	
B-lymphoblastic leukaemia/lymphoma with high hyperdiploidy	2 (11%)	nd	
B-lymphoblastic leukaemia/lymphoma with hypodiploidy	1 (5%)	nd	
B-lymphoblastic leukaemia/lymphoma with ETV6:RUNX1 fusion	1 (5%)	nd	
T-lymphoblastic leukaemia/lymphoma	2 (11%)	190 (19%)	
Age > 35 years (%) (16–76 yo)	13 (68%)	344 (35%)	*p* < 0.05
Male (%)	12 (63%)	619 (62%)	
WBC count > 30 × 10^9^/L	7 (37%)	nd	
Induction chemothrapy			
Cyclophosphamide + Doxorubicin + Vincristine + Dexamethathone	19 (100%)	995 (100%)	
to Methothraxat + Cytosine adabinoside			
Outcome			
Complete remission (CR)	17 (89%)	905 (91%)	
Relapse within one year	8 (42%)	nd	
Progressive disease (PD)	2 (11%)	nd	
Allogenic transplantation	7 (37%)	nd	
Overall survival (OS) at 5 years	8 (42%)	378 (38%)	

Abbreviations: yo: years old; Ph: Philadelphia chromosome; WBC: white blood cell; CR: complete remission; PD: progressive disease, nd: not described. MRC UKALL: the members of the United Kingdom Medical Research Council Adult Leukaemia Working Party.

## Data Availability

The original contributions presented in this study are included in the article. Further inquiries can be directed to the corresponding author(s).

## Patient and Public Involvement

None of the patients were involved in the design of this study. The results of the study will be made known to the study participants on the homepage of our website.

## Abbreviations

The following abbreviations are used in this manuscript:

ALL (AL)	Acute lymphoid leukemia
AML (AM)	Acute myeloid leukemia
CVAD	Cyclophosphamide, vincristine, Adriamycin, dexamethasone
MA	Methotrexate, cytosine arabinoside
OS	Overall survival
IHC	Immunohistochemical staining
KM	Kaplan–Meier
MRP1	Multidrug resistance-associated protein 1
AKR1B10	Aldo-keto reductase family 1 member B10
AKR1B1	Aldo-keto reductase family 1 member B1
AKR1C3	Aldo-keto reductase family 1 member C3
CYP2B6	Cytochrome P450 2B6
ELN	European Leukemia Net
MRC	Medical Research Council
LBCL	Large B-cell lymphoma
TCL	Aggressive T-cell lymphoma
GRP94	Glucose-regulated protein 94
GRP78	Glucose-regulated protein 78
B-ALL	B-cell acute lymphoblastic leukemia
CHO	Cyclophosphamide, Hydroxyl doxorubicin, oncovin
TGFβ1	Transforming growth factor β1
TNFα1	Tumor necrosis factor α1
TNFR	Tumor necrosis factor receptor
PD-1	Programmed cell death-1
PD-L1	Programmed cell death–ligand 1
ENT1	Equilibrative nucleoside transporter 1
MDR1	Multidrug resistance 1
CYP3A4	Cytochrome P450 3A4
TKI	Tyrosin kinase inhibitor
TP	Thymidine phosphorylase
GST	Glutathione sulfate transferase
CR	Complete remission
PD	Progressive disease
M	Months
NS	Not significant
Ph	Philadelphia
NR	not reached
ER	endoplasmic reticulum
UG	Urayasu classification
Significant difference * denotes *p* < 0.05.	
